# Comparison of Weibull and Lognormal Cure Models with Cox in the Survival Analysis Of Breast Cancer Patients in Rafsanjan

**Published:** 2017-02-16

**Authors:** Mina Hoseini, Abbas Bahrampour, Moghaddameh Mirzaee

**Affiliations:** ^1^ Department of Biostatistics and Epidemiology, School of Public Health, Kerman University of Medical Sciences, Kerman, Iran; ^2^ Modeling in Health Research, Institute for Future Studies in Health, Department of Biostatistics and Epidemiology, School of Public Health, Kerman University of Medical Sciences, Kerman,Iran

**Keywords:** Cure Model, breast cancer, Cox regression, Lognormal

## Abstract

**Background:** Breast cancer is the most common cancer after lung cancer and the second cause of death. In this study we compared Weibull and Lognormal Cure Models with Cox regression on the survival of breast cancer.

**Study design:** A cohort study.

**Methods:** The current study retrospective cohort study was conducted on 140 patients referred to Ali Ibn Abitaleb Hospital, Rafsanjan southeastern Iran from 2001 to 2015 suffering from breast cancer. We determined and analyzed the effective survival causes by different models using STATA14.

**Results:** According to AIC, log-normal model was more consistent than Weibull. In the multivariable Lognormal model, the effective factors like smoking, second -hand smoking, drinking herbal tea and the last breast-feeding period were included. In addition, using Cox regression factors of significant were the disease grade, size of tumor and its metastasis (*P*-value<0.05). As Rafsanjan is surrounded by pistachio orchards and pesticides applied by farmers, people of this city are exposed to agricultural pesticides and its harmful consequences. The effect of the pesticide on breast cancer was studied and the results showed that the effect of pesticides on breast cancer was not in agreement with the models used in this study.

**Conclusions:** Based on different methods for survival analysis, researchers can decide how they can reach a better conclusion. This comparison indicates the result of semi-parametric Cox method is closer to clinical experiences evidences.

## Introduction

Breast cancer is one of the most prevalent cancers, particularly in the Eastern Mediterranean. Breast cancer in Iran makes up 16% of all women's cancer^2^. According to WHO, breast cancer is increasing by 1.8 to 2 percent every year^3^. The reason can be the mixture of hormonal, genetic and environmental factors ^4^. Understanding the forewarning death risk of people suffering from breast cancer factors can play a significant role in treatment's and care phases of patients. Although we have had so many researches on specifying factors of these patients and estimating its survival period, we had various results for each part of country^5-6^. Survival analysis is known as a method which is generally used for effectiveness of treatment methods, patient lifetime and detecting effective causes. There are different models of survival analysis which applying them appropriately can lead us to favorable results. For different diseases, researchers have often used Cure Models but in our country researchers have used Cure Models less often for analyzing data to recognize effective factors on survival related to breast cancer.

A survival analysis is based on special hypothesis^7-8^. For example in standard survival analyses (parametric & semi-parametric), the base hypothesis is that all samples will undergo events similar to death within the enough fallow up time. Standard survival analysis does not take into account the fact that the fraction of samples will not experience the expected events or they will be long-term survivors. Thus when analysis of event's time is studied, and some of those societies are immune event or in other words safe, Cure Models are used. In such studies, people were divided into groups of sensitive and insensitive (immune people, safe or with a long term survival). People with long-term survival are immune of expected event. When there are no safe people, mixed Cure Models can be altered to standard survival models^9^. The principal purpose of mixed Cure Models is estimating cured or safe proportion, those who do not experience expected event, and estimating survival role for those who are subjected to expected event (capable people) as well as defining effective factors on these two groups ^9-10^. One of the most significant statistical models in survival analyses is Cox proportional risk model. One of the primary reasons for wide application of Cox model is that this model does not make any assumption about particular distribution on survival time variable^11^. As there are fewer hypotheses in semi-parametric models than parametric models, medical scholars mainly have a tendency to use these models but we should consider that in special situation, when theories of parametric models are set up, these models have more precise estimate than Cox model and present more accurate analysis^12^. One of the most important conditions for applying Cox model is to establish proportional hypothesis of risks. Therefore the objective of this paper is to analyze and compare Weibull and Lognormal Cure Models with Cox regression in survival analysis of breast cancer patients.

## Methods

In this retrospective cohort study we examined 140 breast cancer patients reffered to Ali Ibn Abitaleb Hospital, Rafsanjan, Southestern Iran between 2000 and 2015.

Death of patients because of breast cancer was shown as (failure) and patients who survived by the end of the study were known as (censored). Response variable was the time interval between diagnosis and patient's death, and the end of the study period‏. Independent variables, including tobacco using or being in a close exposure with tobacco users, drinking herbal tea, the duration of last time feeding, number of caesarean, tumor size, grade, body mass index (BMI), tumor metastasis situation, the distance of the patient's location to nearest pistachio orchard, being exposed to agronomic poisons, history of neurological disease, history of hormonal disease, family history of malignant tumor and treatment were taken into consideration.

Recorded data of patients have been collected from non-contagious diseases software and their files in health services, Rafsanjan University health department. Using the data collected, phone calls and face to face interviews, the resulted checklist for each patient was completed, then gathered and labeled data have analyzed by SPSS 22 software. We fitted Cure Models into Stata 14 software. Survival analyses and Cure Models analysis were done and the result of considered variables on patient's survival was analyzed. The significant level was (0.05), and Logit link function was mentioned in the study.

### Statistical analyses

The notion of long- term survival or existence of immune people when there are censored ones in survival data is not a new idea. Boag from England and Berkson and Gage from America are pioneers of this concept ^13-14^. Cure Models in survival analyses are categorized in two groups, mixed and unmixed Cure Models^15^. In mixed Cure Models, it is supposed that the community falls into two groups: people who are at risk and those who are immune. People at risk are those who may encounter death or relapse and sometimes after the beginning of the study they may endure these. The second group is those who are not at risk of death or relapse. The equation mentioned below shows relation between S_0_(t) and the proportion that have long term survival via Cure Model.

^S_τ_^(t)^=p(T_i_>t)=^

^p{T_i_>t│β_i_=1}P(β_i_=1)^

+p{T_i_>t│β_i_=0}P(β_i_=0)

^(1-π)S_0_(t)+π^

This proportion (π) can be a function of variables being analyzed. So those survival parameters which effect long-term survival can be traced. This section is called long term survival model. The second part of model shows the proportion (1-π) of patients who are at the risk of death or relapse. It assists to find the effected factors on long term survival and it is feasible to analyze the existence of patients with long term survival and the sufficiency of study time via the use of statistical tests^16^.

This hypothesis is compiled in this form H0:π=1. It indicates that all the people may experience death or relapse and there is no cure fraction. There is a model proposed by Maller and Zhou which is based on I.I.D censored model and it' is on the basis of exponential and uniform distribution for being censored. In order to accept or reject stated hypothesis, critical values have been set in a table^9^. Another hypothesis which should be analyzed before using Cure Models is sufficiency of follow-up time. Maller and Zhou via sampling have prepared some tables under the same situation as in previous test (I.I.D being of censor time, exponential and uniform distribution of censoring time). For doing the test, we should apply the following steps:

1. Finding the latest observed failure (t_n_*)
2. Finding the latest observed censoring time (t_n_)
3. Calculating N_n_ with counting the number of failures betweent_n_* and ( 2t_n_*-t_n_) :

‏ N_n_=( 2t*_n_-t_n_ , t*_n_)

4. calculatingqn with division of N_n_ to the number of study sample size.
5. At last the number of q_n_calculated in previous steps was compared to critical value related to the study sample size and suitable *β* and *μ* of sampling tables of Maller and Zhou that *β* is parameter of uniform distribution and *μ* is parameter of exponential distribution.

As it was expressed for using Cox semi-parametric model we are supposed to analyze the establishment of necessary precondition. The necessary prerequisite of using this model is that relative risk in being compared groups should be fixed during the time. For doing this pre-test, Stata11 software was applied. In this study due to comparing the use of Weibull and Lognormal Cure Model, AIC criterion was used. AIC criterion was proposed in 1974, to measure the excellence of fitness model ^9, 17^. This criterion measures the extent of interaction between the complexity of model and suitable fitness of model. For Weibull and Lognormal models this criterion is computed via the use of the formula in which p which is the number of accessible parameters in k model is a fixed factor that its value is relevant to the type of used model and for Lognormal and Weibull model, this factor is 2^8^. The smaller the amount of AIC the more useful the model is:

AIC= -2log (likelihood) +2(p+k)

For Cure Models logit link function was taken into consideration and the whole variables were separately plotted in Weibull and Lognormal model. Log-normal was chosen as a more consistent model than Weibull as its AIC was less than the other model in 85% of variables. The significant results of variables on short and long-term survival as well as estimate of patients with long-term survival for the chosen model were reported. Then via the use of backward method, multivariable Cure Model was fitted on data. Then after choosing effective variables of Cox model, multivariable Cox model was fitted via the use of backward method, and eventually we chose the best model between Cox and Cure Model based on AIC.

Since the data in this study were insufficient, we used Cross validation at 110 times to support the results. Then RMSE (t) of Cross validation Compared with the RMSE (t) of Cox model and Kaplan Meier Which can be calculated via applying the following formula:

$$\text{RMSE (t) =}\sqrt{\frac{1}{n}\sum_{i=1}^{n}\sum {\left({\hat{s}}_{i}(t)-s_{i}(t)\right)}^{2}}$$

If RMSE (t) of Cross validation is close to RMSE (t) of Cox model and Kaplan Meir, The validity of the results will be confirmed ^18^. In addition, ROC curve was extended to survival data as the beast model and AUC for each variables was calculated ^19^.

## Results

**Figure 1:**
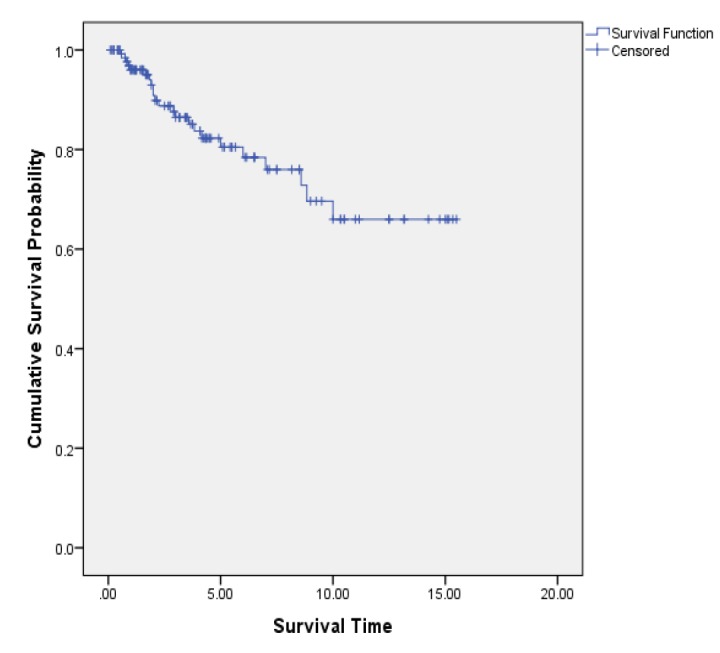
Plot of probability of survival patients and test of cure fraction existence hypothesis and sufficient follow up duration of patients

The study was conducted on 140 patients in a 96 months period (15 years and 6 months).The mean age of patients at the time of entering the study was 47.1 and its median was 46.2 with a maximum age of 89.8 and a minimum age of 24.3. The Kaplan Meier diagram has been shown in Figure 1. As it is clear in the diagram after 10 years from the beginning of study, the curve stabilized for about five years. From this diagram we can understand that there are people with long-term survival. Patients who have had Surgery, chemotherapy, radiotherapy and hormone therapy together had high likelihood of cure 69% (95%CI: 0.65, 0.73).The mean survival of patients was 11.93 with 95% confidence interval of [95% CI: 10.67, 13.21]. The percentage of Long-term survival people was 0.64 with 95 % confidence interval of [95% CI: 0.57, 0.71]. The possibility of 5, 10 and 15 years survival of patients were about 0.8, 0.65 and 0.65, respectively.

The testing of patient's existence with long-term survival, using Kaplan Meier method was analyzed. By considering these data and via the use of Maller and Zhou table we accepted the hypothesis of long-term survival patients or the existence of cured people and sufficiency of fallow up time.

The AIC criterion for the two mixed Cure Models, Weibull and Lognormal, was reported as independent variables separately. AIC of Lognormal distribution in approximately 0.85 of variables are smaller than that of Weibull. It means that Lognormal distribution has attained better fitness than the Weibull distribution. Multivariable significant results for patients with long-term survival have just reported for Lognormal distribution. Therefore we entered all the variables to Cure Model of Lognormal and variables whose p-value was less than or equal top 0.2 were selected for fitting multivariable Lognormal model.

In Table 1, the results of Lognormal multivariable model are presented. Table 2 also is related to multivariable semi- parametric Cox model which is given via the use of backward method. Comparing multivariable cure Lognormal model with multivariable Cox model:

**Table 1:** Lognormal multivariable Cure Model findings

**Variables**	**OR (95% CI)**	***P*** ** value**
**Long term survival**		
Smokers and second –hand smokers		
No	1.00	
Yes	0.08 (0.01,0.84)	0.036
Drinking herbal tea		
No	1.00	
Yes	0.01 (0.01, 0.98)	0.049
The duration of last time feeding(month)	3.56(1.18,10.74)	0.024
**Short term survival**		
Number of caesarean	1.95(1.06,3.58)	0.032
Tumor size(cm^3^)	0.98(0.97,0.99)	0.001
The duration of last time feeding(month)	0.90(0.85,0.95)	0.001

**Table 2:** Regression results of multivariable Cox in breast cancer patients

**Variables**	**HR**	**95% CI**	***P*** ** value**
Grade^a^			
2	1.00		0.053
3	4.72	(0.67,33.21)	0.119
4	11.53	(1.40,97.13)	0.026
Tumor size(cm^3^)	1.05	(1.04,1.06)	0.010
Tumor metastasis situation			
No	1.00		
Yes	10.97	(1.56,77.16)	0.016

^a^ There was no patient in grade 1

In the previous sections we studied the validity of variables on the long-term survival in a single variable method. In this part the importance of variables in multivariable type is studied. First each variable was entered individually into Lognormal Cure Model and then the results were analyzed. Those variables which had a p-value less than or equal to 0.2 entered both into long and short-term survival of multivariable Lognormal model .After that using backward method those variables whose p-values were less than or equal to 0.05, were put in the final model.

Table1 shows the final model with its coefficients, standard error, significant coefficient and confidence interval of 95% for coefficient. The number of caesarean, the tumor size and the duration of the last breast-feed variables had significant effect on short-term survival.

For instance, with each unit increase in tumor size, the mean survival time will be multiplied by 0.98. Tobacco use or being exposed to tobacco, herbal tea and the duration of last breast-feed had significant effect on the long-term survival. Based on the point that the variable whose OR is near or equal to 1 is an ineffective factor, a variable whose OR is more than 1 is a risk factor and the one with OR less than 1 is considered as a protection factor. Thus it will be possible to define the long-term survival part of the model. Cox multivariable regression results in a backward method with presenting the regression coefficient, coefficient standard error, significant coefficient, relative danger and the confidence interval of %95 of relative risk are presented in table 2.

Grade, the size of tumor and tumor metastasis situation variables are related to the duration of survival. Classified grade variable had better fitness, so it is entered to the model in a classified form. Data show (table2) that Grade variable, the size of tumor and tumor metastasis situation have significant effect on patients survival.

The size of tumor is calculated in cubic centimeter. Tumor metastasis variable with the coefficient of 2.4 and p-value of less than 0.05 increases risk rate to 10.97 times. It also increases the risk function and decreases the patient's survival. It means that patients who have experienced metastasis are 10.97 times more in death risk compared to patients who haven't experienced that. AIC for Cox model and for log-normal model was 134.67 and 165.31, respectively showing that the AIC for Cox model is less than that of for log normal model. So Cox model is better than the other model. Moreover the findings showed that the results of the Cox model are closer to clinical observations than those of Lognormal model.

**Figure 2:**
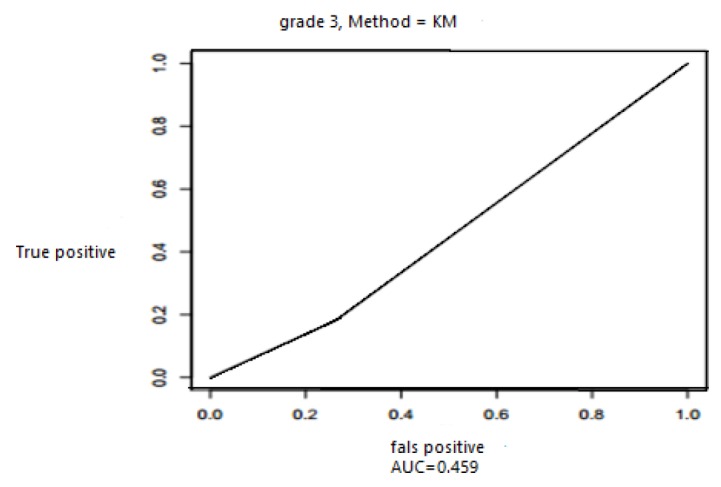
Plot of ROC curve extended to survival data (Cox model) for grade 3

Using Cross validation RMSE (t) was 0.4059, while with Cox model and Kaplan Meir was it was 0.22.These findings make this study valid. The results of extending ROC curve to survival data (Cox model) were as follows: AUC for grade (g3, g4), tumor metastasis situation and tumor size respectively were 0.46, 0.45, 0.40 and 0.44. In Figure 2, ROC curve for grade 3 (g3) is presented.

## Discussion

In this study, necessary hypotheses for using Cure Models in survival analysis of breast cancer patients were analyzed and after that the use of two cures models, Lognormal and Weibull, in patients' survival analyses were compared with each other. These data later using Cox regression were analyzed. Because Rafsanjan is surrounded by pistachio orchards and pesticides used for agricultural purposes, people of this city are exposed to those pesticides so that their harmful consequences. In this study we determined the effect of the pesticides on breast cancer.

Most of researchers have used standard survival methods for analyzing data related to patients with breast cancer. However, in Iran less often Cure Models has been used. Studies have used Cox model to determine the efficient factors on survival risks in breast cancer patients. In Shiraz patients in primary levels of disease experience higher rates of long-term survivals and have more hopes for life ^20^.

Metastasis being diagnosed at its earlier stage can decrease patient's overall survival and help the process of relapse ^21^.

In another study on long and short-term survival of renal allograft patients, according to the results Cure Models may potentially enhance the understanding of factors effecting long and short-term survival ^22^.

Akhlaghi et al. in their study argued that Cure Modeling not only is beneficial in separation of long and short-term survival patients but also can highlight distinct elements on them and present more accurate description of the data ^23^.

For predicting the survival of patients with breast cancer, the use of artificial neural network, is more suitable tool than logistic model and through early detection of disease at younger age, and applying necessary treatment, survival of these patients can be enhanced ^24^.

In another study, the classification and regression trees model produced a decision tree with 17 nodes, 9 of which were working in relationship with a set of rules. The rules were clinically significant. From the if-then format, they showed that stage played an important role in breast cancer survival prediction. Sensitivity scored the highest (93.5%) and specificity was the lowest (53%), ^25^.

According to another study, the survival rates decreased as the age of patients having the cancer increased. By employing censored quintile regression model they showed that significant factors change affecting the median and quantities of breast cancer ^26^.

Rahimzadeh et al. in their study found relative survival rates in 1-year, 3-year and 5-year as 97%, 89% and 74%. ^27^they also highlighted that age was not as significant as other variables such as phase of the breast cancer and the carcinogenic nature oh the disease. Thus those were considered as cure rate indexes.

Jafari-Koshki et al. found that free survival was more than 6 years. The significant variables in the their study were the number of lymph node and progesterone receptivity mode, with an indirect and a positive effect on breast cancer, respectively. Approximately half of patients were eventually cured. The Weibull model had a little better performance than log-logistic ^28^.

In a study conducted to find the more suitable model to determine the significant factors on breast cancer, Model-Based Recursive Partitioned was consider as a potentially useful model to process sophisticated mixture Cure Models. The 5-year survival rate, median life time, and the mortality rate were 68.5%, 9.02 and 36.73%, respectively. The patients selected ranged from 22 to 79 year of age when diagnosed with breast cancer. (SD=46.1, median =45) ^29^.

To apply Cox model it is necessary to have the hypotheses lodgment, one is being exposed to risk for all patients. In this study there were patients with long-term survival and Weibull and Lognormal Cure Models were employed. As patients will gradually die (by the end of the study), we used Cox risks model. The comparison of Weibull and Lognormal Cure Model with the use of AIC showed that Lognormal model has a better fitness on most variables. So we reported the significant results of multivariable for patient's with-long term survival for Lognormal distribution.

Also Cox risk model results in multivariable form have been offered. Different results show that if researchers do not pay enough attention to choosing their analysis method and do not employ the clinical experiences and biological evidences of their study field, they may gain misleading results. Also existence of adequate data and suitable follow-up time duration are effective factors for reaching reliable results in Cure Models.

## Conclusions

Cure Model can separate short and long-term survival of patients and determine the effective factors on them .This ability can lead to more accurate interpretation of survival data and thus a greater ease in authorities decision-making in the field of public health while standard survival methods only present effective factors on general survival of patients.

## Acknowledgements

The researchers of this study appreciate the health deputy of Rafsanjan University of Medical Sciences and also administrative director of Ali Ibn Abitaleb hospital for cooperating in collecting part of patients' data.

## Conflict of interest statement

The authors confirm that they have no conflict of interest to declare.

## Funding

This study is financially supported by research team.

## Highlights

- The most important affected factor on breast cancer are smoking, breast-feeding, tumor size and grade
- The results showed that the Lognormal is better than Weibull according to AIC.
- Log-normal and Weibull Cure and Cox models are compared. Log-normal and Weibull Cure and Cox models are compared
- The Cox model was closer to clinical observations.
